# Neurodegenerative Diseases: Might *Citrus* Flavonoids Play a Protective Role?

**DOI:** 10.3390/molecules21101312

**Published:** 2016-09-30

**Authors:** Santa Cirmi, Nadia Ferlazzo, Giovanni E. Lombardo, Elvira Ventura-Spagnolo, Sebastiano Gangemi, Gioacchino Calapai, Michele Navarra

**Affiliations:** 1 Department of Chemical, Biological, Pharmaceutical and Environmental Sciences, University of Messina, viale Annunziata, Messina I-98168, Italy; scirmi@unime.it (S.C.); nadiaferlazzo@email.it (N.F.); 2 Department of Health Sciences, University “Magna Graecia” of Catanzaro, Catanzaro I-88100, Italy; gelombardo@unicz.it; 3 Department of Biotechnology and Legal Medicine, University of Palermo, Palermo I-90127, Italy; elvira.ventura@unipa.it; 4 Department of Clinical and Experimental Medicine, University of Messina, Messina I-98125, Italy; gangemis@unime.it; 5 Institute of Applied Sciences and Intelligent Systems (ISASI), National Research Council (CNR), Pozzuoli I-80078, Italy; 6 Department of Biomedical and Dental Sciences and Morphofunctional Imaging, University of Messina, Messina I-98125, Italy; gcalapai@unime.it

**Keywords:** *Citrus*, flavonoids, neurodegeneration, neurodegenerative disorders, nutraceutical

## Abstract

Neurodegenerative diseases (ND) result from the gradual and progressive degeneration of the structure and function of the central nervous system or the peripheral nervous system or both. They are characterized by deterioration of neurons and/or myelin sheath, disruption of sensory information transmission and loss of movement control. There is no effective treatment for ND, and the drugs currently marketed are symptom-oriented, albeit with several side effects. Within the past decades, several natural remedies have gained attention as potential neuroprotective drugs. Moreover, an increasing number of studies have suggested that dietary intake of vegetables and fruits can prevent or delay the onset of ND. These properties are mainly due to the presence of polyphenols, an important group of phytochemicals that are abundantly present in fruits, vegetables, cereals and beverages. The main class of polyphenols is flavonoids, abundant in *Citrus* fruits. Our review is an overview on the scientific literature concerning the neuroprotective effects of the *Citrus* flavonoids in the prevention or treatment of ND. This review may be used as scientific basis for the development of nutraceuticals, food supplements or complementary and alternative drugs to maintain and improve the neurophysiological status.

## 1. Introduction

The increase of the lifespan in populations of developed countries is leading to a rise in the incidence of age-related illnesses such as neurodegenerative diseases (ND). ND are a heterogeneous group of chronic and untreatable conditions that, in terms of human suffering and economic cost for society, represent the fourth highest source of overall disease burden in high-income countries. ND result from the gradual and progressive degeneration of the structure and function of both the central nervous system (CNS) and the peripheral nervous system (PNS), characterized by deterioration of neurons and/or myelin sheath, sensory information transmission disruption and movement control.

It is known that oxidative stress and chronic inflammation play an important role in ND. Free radicals represent a common denominator of many oxidative-based diseases, including ND, although they are normally and continuously produced in CNS, acting as important cellular messenger. However, excessive levels of reactive oxygen species (ROS) or reactive nitrogen species (RNS) are involved in numerous neuroinflammatory processes. In physiological conditions, the levels of free radicals are tightly regulated by a complex antioxidant defense system including both enzymatic and non-enzymatic antioxidants. Disruption of the delicate oxidant/antioxidant balance between the production and removal of oxidizing chemical species can lead to oxidative stress triggering cell damage. This may be due to an excessive production of ROS and RNS and/or at a reduced efficiency of the physiological antioxidant defense systems. Moreover, because of their high metabolic activities and low antioxidant defense capability, neural cells in brains are more vulnerable to oxidative stress, especially those of the aging brain. Furthermore, cytokines released by the activated glial cells amplify the free radicals-induced damage responsible for uncontrolled proteolysis, DNA mutagenesis, lipid peroxidation and cell death.

Currently there is no effective treatment for ND, and the marketed drugs are mainly symptom-oriented, albeit with many side effects, limited efficacy and partial capability to inhibit disease progression. Therefore, in order to develop novel preventive strategies or co-adjuvant therapy for ND, within the past decades, a great number of natural medicinal plants has gained attention as potential neuroprotective agents [1]. Moreover, an increasing number of studies have suggested that dietary intake of vegetables and fruits can prevent or delay the onset of ND [2,3]. These properties might be due to the presence of polyphenols, an important group of phytochemicals that are abundantly present in fruits, vegetables, cereals and beverages [4]. The main class of polyphenols is flavonoids which display a remarkable spectrum of biological activities, including antioxidant, free radical scavenger, metal ions chelating, vasoprotective, hepatoprotective, anti-inflammatory, anti-cancer and anti-infective [5,6]. Main dietary sources of flavonoids are fruits, especially *Citrus* fruits, vegetables, fruit juices, tea, coffee, and red wine [7]. Several lines of evidence have demonstrated the beneficial effects of *Citrus* fruits in the context of numerous pathologies, including ND, inflammation, cardiovascular diseases, dyslipidemia, diabetes, allergy, immune system diseases and cancer [8,9,10,11,12,13,14,15,16,17,18,19,20,21,22,23,24].

Our review is an overview on the scientific literature concerning the neuroprotective effects of the *Citrus* flavonoids in the prevention or treatment of ND.

## 2. Focus on Neurodegenerative Disorders

ND are incurable conditions due to the progressive nervous system dysfunction caused by degeneration and loss of nerve cells for reasons that have not yet been fully understood. Today, a growing number of people worldwide are affected by ND, characterized by deterioration in emotional control, social behavior and social communication. ND exist in many forms, such as Multiple Sclerosis, Alzheimer’s, Parkinson’s, Huntington’s, human prion and motoneuron diseases.

Alzheimer’s disease (AD) is a debilitating ND classified as the major subtype of dementia [25], and is characterized by progressive loss of memory. It results in decline in cognitive and behavioral functions like memory, thinking and language skills [26]. The hallmarks of AD are (i) the accumulation of amyloid-beta (Aβ) peptide in the brain; (ii) the presence of neurofibrillary tangles (NFTs) containing hyper-phosphorylated tau fragments and (iii) the loss of cortical neurons and synapses [27,28]. It is also known that the innate immune system activation plays a relevant role in the age-related ND, including AD. Microglia are innate immune cells in the CNS that mediate inflammatory responses to injury and pathogens by releasing pro-inflammatory cytokines that amplify and aggravate inflammation throughout the brain. These pro-inflammatory factors may induce degeneration of normal neurons through upregulation of nuclear factor kappa B (NF-κB), mitogen-activated protein kinase (MAPK), and c-Jun N-terminal kinase (JNK) [29].

Parkinson’s disease (PD) is the second common neurodegenerative disorder and its incidence is increasing among people over the age of 60 years [30]. It is consistently higher in men than in women [31]. Pathologically, it is characterized by the progressive and diffuse loss of dopaminergic neurons in the substantia nigra and the accumulation of Lewy bodies (inclusions containing α-synuclein) in nerve cells. The major clinical symptoms are tremor at rest, rigidity, bradykinesia and postural instability. It has been suggested that activated microglia may be beneficial to the host in the early phase of neurodegeneration but excessive activation of microglia leads to the elevated expression of pro-inflammatory mediators such as tumor necrosis factor alpha (TNF-α), interleukin 1 beta (IL-1β), interleukin-6 (IL-6) and interferon gamma (IFN-γ) [32], that induce the degeneration of substantia nigra pars compacta dopaminergic neurons [29,33]. Furthermore, the release of these pro-inflammatory mediators can activate astrocytes that participate to the neuroinflammatory processes linked to PD [34].

Huntington’s disease (HD) is a hereditary neurological disorder inherited as an autosomal dominant trait [35] caused by an expanded polyglutamine tract in the N-terminal region of mutant huntingtin [36]. HD usually occurs in early middle life even if there is an uncommon juvenile form [37]. It is an illness that recurs with abnormal movements together with psychiatric symptoms including psychosis, depression, and obsessive-compulsive disorder and progressive cognitive impairment [38,39,40]. In the early stage of disease, neuronal loss occur preferentially in the striatum, while in the later stages the extensive neurodegeneration happens in a variety of brain regions [36,41].

Human prion diseases are fatal neurodegenerative disorders which include Kuru, Creutzfeldt-Jakob disease, Gerstmann-Sträussler-Scheinker syndrome, and fatal familial insomnia [42]. Prion diseases result from the conformational conversion of a normal cellular prion protein (PrP^C^) into an abnormal misfolded pathological form (PrP^Sc^). Its accumulation in the CNS resulted in progressive neuronal degeneration and vacuolation [43].

Motor neuron disease (MND) is a neurodegenerative condition, among which the most common is the amyotrophic lateral sclerosis (ALS). MND affects both brain and spinal cord, and is due to the degeneration of motor neurons, that in turn causes muscle weakness. The major clinical symptoms include muscle weakness, wasting, cramps and stiffness of arms and/or legs, problems with speech and/or swallowing or, more rarely, with breathing problem [44]. The etiopathogenesis of MND is unknown and the aim of the cure is to maintain functional ability and enabling MND patients to live life as fully as possible [45].

Multiple sclerosis (MS) is a CNS chronic inflammatory disease that is caused by autoimmune-mediated loss of myelin and axonal damage. Its etiopathogenesis is poorly understood. MS is the most common neurological disorder affecting young adults, with a total of 2.5 million people in worldwide [46]. It causes a range of relapsing symptoms during the early phase of the disease, but becomes more persistent and less amenable to treatment at later stages. The research focused on identifying treatments that slow progression of neurodegeneration and also restore myelin of affected CNS regions [47].

## 3. *Citrus* Flavonoids

The genus *Citrus* belongs to the family Rutaceae, subfamily Aurantioideae, tribe Citreae, subtribe Citrinae. According to statistics of Food and Agriculture Organization of the United Nations (FAO), *Citrus* species are grown in more than 140 countries. China, Brazil, USA, India, Mexico, and Spain are the world’s leading *Citrus* fruit-producing countries, representing close to two-thirds of global production (FAO STAT) [48].

**Figure 1:**
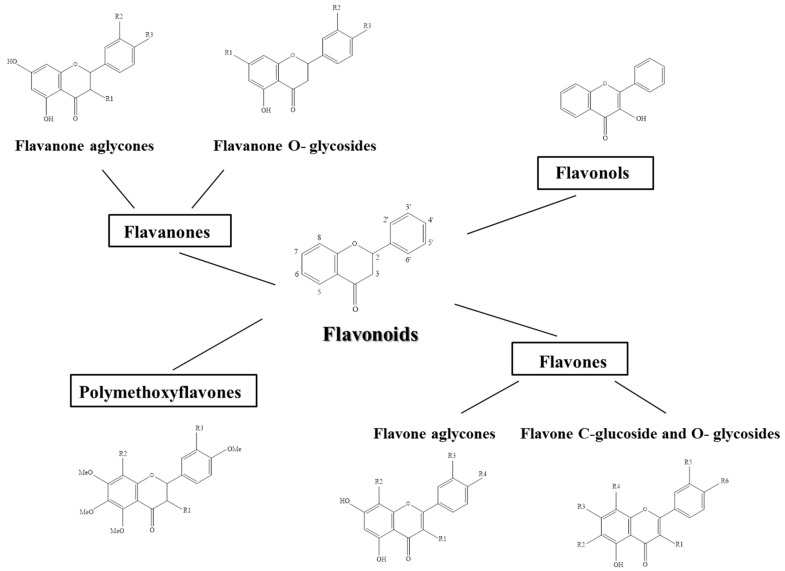
Chemical structure of *Citrus* flavonoids subclasses.

The basic structural feature of flavonoid is 2-phenyl-benzo-γ-pyrane nucleus, contains a C6−C3−C6 heterocyclic skeleton, consisting of two benzene rings linked through a heterocyclic pyran ring. Based on the oxidization of the heterocyclic (C3) ring, *Citrus* flavonoids can be divided in flavanones, flavonols, flavones, and polymethoxiflavone (Figure 1). Anthocyanins are considered as metabolites of flavones and are present only in blood oranges. Structurally, they derived from pyran or flavan. Flavonoids are mainly present in plants as glycosides, while aglycones (the forms lacking sugar moieties) occur less frequently. Therefore, the large number of flavonoids is a result of many different combinations of aglycones and sugars, mainly d-glucose and l-rhamnose, bounded the hydroxyl group at the C-3 or C-7 position.

**Figure 2:**
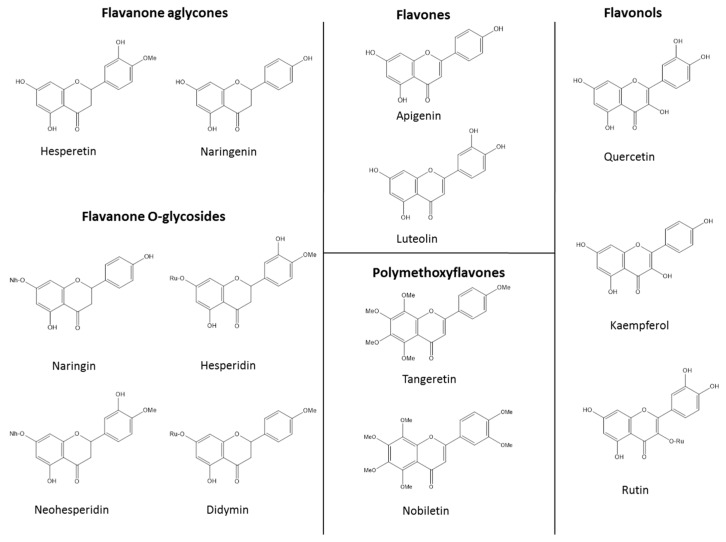
Molecular structure of *Citrus* flavonoids discussed in this review. Rutinose (Ru), neohesperidose (Nh), methoxy (Me).

Flavanones comprise approximately 95% of the total flavonoids. Their concentration depends on the age of the plant, and the highest levels are detected in tissues, showing pronounced cell divisions [49]. They are present in both the glycoside and aglycone forms. Glycosylation occurs at position 7 either by rutinose or neohesperidose. The most important flavanones present in the aglycone forms are naringenin and hesperetin. Among flavanones with neohesperidose (rhamnosyl-α-1,2 glucose), naringin, neoeriocitrin, neohesperedin and poncirin are the most abundant. Hesperidin, narirutin, eriocitrin and didymin are the main flavanone with rutinose (rhamnosyl-α-1,6 glucose). Luteolin and diosmetin are the most present flavones in the aglycone form, while diosmin and neodismin represent the major flavones in the rutinosides and neohesperidosides forms, respectively [50]. Flavonols are the 3-hydroxy derivatives of flavones. Glycosylation occurs preferentially at the 3-hydroxyl group of the central ring. The most common flavonol aglycones are quercetin and kaempferol, while rutin and rutinosides are the main in the glycosidic form. Even if present in smaller quantities, the most common polymethoxiflavones are tangeretin and nobiletin. The chemical structures of *Citrus* flavonoids discussed in this review are presented in Figure 2.

### 3.1. Flavanones

In Table 1 are summarized the studies reporting the neuroprotective activity of *Citrus* flavanones.

#### 3.1.1. Naringin

Naringin is a major flavanone glycoside in *Citrus* fruits and it is considered a neuroprotective agent mainly because its anti-apoptotic [75,76] and anti-oxidant activities [77], together with its capability to induce neurotrophic factors such as brain-derived neurotrophic factor (BDNF) and vascular endothelial growth factor (VEGF). Recently, Leem and coworkers (2014) evaluated the effect of naringin in a neurotoxic model of PD in vivo. They found that the flavanone could prevent the degeneration of the nigrostriatal dopaminergic (DA) projection by increasing the level of glia-derived neurotrophic factor (GDNF) in nigral DA neurons, with activation of mammalian target of rapamycin complex 1 (mTORC1). Furthermore, they observed that naringin could attenuate the rise of TNF-α induced by 1-methyl-4-phenylpyridinium (MPP^+^) in microglia, indicating its anti-inflammatory activity in CNS [51]. Very recently, the same research group evaluated the effects of pre- or post-treatment with naringin in a 6-hydroxydopamine (OHDA)-treated mice [52], suggesting that it protected the nigrostriatal DA projection from 6-OHDA-induced neurotoxicity by both the activation of mTORC1 and the inhibition of microglial activation [51]. In 2012, Gopinath and Sudhandiran investigated on the neuroprotective effect of naringin on 3-nitropropionic acid (3-NP)-induced neurodegeneration [53]. The 3-NP is a natural environmental toxin that causes selective neuronal degeneration in the striatum, reproducing in animal models the brain lesions observed in HD patients [78]. The experimental protocol required that rats received naringin orally (80 mg/kg body weight/day) 1 h prior to the intraperitoneal injection of 3-NP (10 mg/kg body weight/day) for 2 weeks (the time to develop neurodegeneration). The Authors observed that naringin can mitigate 3-NP-induced neurodegeneration, through the enhancement of antioxidant enzyme gene expressions via nuclear factor E2-related factor 2 (Nrf2) activation, thus modulating oxidative stress and inflammatory responses.

In the last decade, flavonoids have been used to reduce neurotoxic effects of aluminum (Al) chloride (AlCl_3_) in rats. In this field, Prakash and collaborators (2013), explored the possible role of naringin against Al-induced cognitive dysfunction and oxidative damage in rats. The Authors observed that chronic administration of naringin (40 and 80 mg/kg) for six weeks significantly improved cognitive performance and attenuated mitochondria oxidative damage, acetyl cholinesterase activity, and Al concentration in Al-treated (100 mg/kg) rats [54]. The same authors showed that treatment with naringin (40 and 80 mg/kg/day) for 25 consecutive days beginning 4 days prior to colchicine (15 μg/5 μL) injected intracerebroventricularly significantly improved the cognitive performance and attenuated oxidative damage in colchicine-treated rats [55].

#### 3.1.2. Hesperidin

Hesperidin, a flavonoid that is particularly abundant in oranges and lemons, exerts anticarcinogenic, antihypertensive, antiviral, antioxidant and antiinflammatory effects [79]. In addition, hesperidin can cross the blood-brain barrier [80] and can protect the neurons against various types of insult associated with neurodegenerative diseases, including AD, PD, and HD. A study performed by Antunes and collaborators (2014) was aimed to evaluate the role of hesperidin in an animal model of PD induced by 6-OHDA. The Authors demonstrated that hesperidin (50 mg/kg), administrated for 28 days after an intracerebroventricular injection of 6-OHDA, was effective in preventing memory impairment and depressive-like behavior in mice. Furthermore, in the striatum of aged mice, hesperidin attenuated the 6-OHDA-induced reduction in (i) glutathione peroxidase (GPx) and catalase (CAT) activity, (ii) the total reactive antioxidant potential and (iii) the dopamine levels. Finally, the flavanone mitigated both the increased levels of ROS and the activity of glutathione reductase induced by 6-OHDA [56].

The implications of nitric oxide (NO) in a variety of neurodegenerative diseases suggests a potential role of flavonoids in HD and other oxidative-based disorders. Menze et al., (2012) investigated the potential effect of hesperidin on 3-NP-induced behavioral, neurochemical, histopathological and cellular changes. They showed that pretreatment with hesperidin (100 mg/kg) ahead of 3-NP (20 mg/kg) for 5 days prevented any changes of locomotor activity or prepulse inhibition, slightly increased malondialdehyde (MDA) levels and reduced inducible nitric oxide synthase (iNOS) positive cells as well as CAT activity in cortex, striatum and hippocampus evoked by 3-NP [57].

One of the most distinctive neuropathological characteristics of AD, are the senile plaques of aggregates Aβ peptide. Moreover, besides the well-known Aβ aggregation, neuro-inflammation also plays a pivotal role in the etiopathogenesis of this multifactorial disorder [81]. Li and coworkers (2015), evaluated the potential therapeutic effect of hesperidin on behavioral dysfunction, Aβ deposition and neuro-inflammation in the transgenic APP/PS1 mouse, a useful model of cerebral amyloidosis for AD. Hesperidin (100 mg/kg body weight) was orally given to the mice for 10 days. It recovered deficits in non-cognitive nesting capability and social interaction and attenuated Aβ deposition, plaque associated amyloid precursor protein (APP) expression, microglial activation and TGF-β1 immunoreactivity in both cerebral cortex and hippocampus of APP/PS1 mice [58]. The same dose of hesperidin (100 mg/kg) administrated for 16 weeks to three-month-old APPswe/PS1dE9 transgenic mice reduced their learning and memory deficits, improved locomotor activity and increased glycogen synthase kinase-3β (GSK-3β) phosphorylation, anti-oxidative defense and mitochondrial complex I–IV enzymes activities [58]. However, there was not observed obvious change in Aβ deposition [59]. Hesperedin was effective also in the experimental model of intracerebroventricular streptozotocin (ICV-STZ)-induced sporadic dementia of Alzheimer’s type (SDAT) [60]. Indeed, pretreatment with hesperidin (100 or 200 mg/kg/day orally for 15 days) improved memory consolidation process possibly through modulation of acetylcholine esterase activity (AChE) in mice injected bilaterally with single dose of ICV-STZ (2.57 mg/kg body weight each side). Moreover, hesperidin decreased neuronal cell death by reducing the overexpression of pro-inflammatory mediators like NF-κB, iNOS, cyclooxygenase-2 (COX-2) and attenuated astrogliosis [60].

One of the key factors in the progression of neurodegenerative diseases is the deregulation of metal ion homeostasis, such as Al, a major risk factor for the AD [82]. Thenmozhi and coworkers (2015) evaluated the protective effect of hesperidin on AlCl_3_ induced neurobehavioral and pathological changes in Alzheimeric rats. Orally administration of hesperidin (100 mg/kg) along with AlCl_3_ injection (100 mg/kg) for 60 days, significantly reduced the Al concentration in hippocampus and cortex, the acetylcholinesterase (AChE) activity, the APP expressions, the levels of both Aβ_1–42_ and its synthesis-related molecules (β and γ secretases). Moreover, hesperidin significantly attenuated the behavioral impairments caused by AlCl_3_ and preserved the normal histoarchitecture pattern of the hippocampus and cortex, as observed by histopathological studies [61]. Very recently, with the same experimental model Thenmozhi et al., (2016) showed that hesperidin prevented oxidative stress and apoptosis induced by AlCl_3_ compared to control group [62].

Excitotoxicity is considered one of the constitutive components of the neurodegenerative diseases pathogenesis [83] caused by excessive release of aminoacids such as glutamate, a crucial excitatory neurotransmitter in the mammals CNS. This provokes the overstimulation of glutamate receptors which leads to an overload of intracellular Ca^2+^, generation of free radicals and subsequent neuronal cell death [84]. Chang and coworkers (2015) evaluated the potential role of hesperidin in neurotoxicity induced by glutamate release in rat hippocampus. They observed that hesperidin (IC_50_ 20 μM) inhibited both the release of glutamate and the elevation of cytosolic free Ca^2+^ concentration evoked by 4-aminopyridine (4-AP) in rat hippocampal nerve terminals (synaptosomes). Furthermore, in hippocampal slice preparations, whole-cell patch clamp experiments showed that hesperidin reduced the frequency of spontaneous excitatory postsynaptic currents without affecting their amplitude, indicating the involvement of a presynaptic mechanism. In addition, the Authors observed that pre-treatment with hesperidin (10 or 50 mg/kg) attenuated the rise of extracellular glutamate and the neuronal loss in the hippocampal CA3 area caused by the intraperitoneal injection of kainic acid (KA; 15 mg/kg) [63].

#### 3.1.3. Hesperetin

Hesperetin is a flavanone abundant in *Citrus* fruit and juice, and represents the major circulating aglycone metabolite of hesperidin. Hesperetin displays several biological properties, such as antioxidant, neuroprotective and anti-inflammatory activities.

Experiments performed in vitro showed that 1 μM hesperetin has neuroprotective effects against H_2_O_2_-induced cytotoxicity in PC12 cells [68], and that much lower concentrations both inhibit H_2_O_2_-induced apoptosis and counteract staurosporine-induced cell death in primary cortical neurons (0.01 μM/L and 300 nM, respectively) [66,67], suggesting its potential role in the intervention for neurodegenerative diseases.

In vivo, Choi and Ahn (2008) observed that hesperetin (10 or 50 mg/kg body weight) inhibited biomarkers of oxidative stress, such as the thiobarbituric acid-reactive substance (TBARS) and carbonyl, in the brains of mice, and activated both CAT and superoxide dismutase (SOD). Moreover, hesperetin increased the reduced glutathione (GSH)/oxidized glutathione (GSSG) ratio, the glutathione peroxidase (GSH-px) and the glutathione reductase (GR) activities [64]. Very recently, Kiasalari and coworkers (2016), evaluated the protective effect of hesperetin against 6-OHDA-induced striatal lesion and have explored some underlying mechanisms including apoptosis, inflammation and oxidative stress. They administrated hesperetin (50 mg/kg/day) for 1 week at intrastriatal 6-OHDA-lesioned rats. They observed that hesperetin reduced the rotational asymmetry induced by apomorphine, as well as the latency and the total time on the narrow beam task. It also decreased striatal malondialdehyde and increases both striatal CAT activity and GSH content. Moreover, hesperetin lowered striatal level of glial fibrillary acidic protein (GFAP) and increased Bcl_2_ [65]. Finally, hesperetin treatment was also capable to mitigate nigral DNA fragmentation and to prevent loss of SNC dopaminergic neurons [65].

#### 3.1.4. Neohesperidin

Neohesperidin is a flavanone glycoside found in *Citrus* fruits. Hwang and coworkers (2008) demonstrated the protective effects of pretreatments (6 h) with neohesperidin, hesperidin and hesperetin (0.8, 4, 20, and 50 μM) on H_2_O_2_-induced (400 μM, 16 h) neurotoxicity in PC12 cells by scavenging ROS, attenuating the elevation of intracellular free Ca^2+^, preventing membrane damage and increasing CAT activity. Furthermore, neohesperidin attenuated both the decrease of mitochondrial membrane potential and the increase of caspase-3 activity evoked by H_2_O_2_ [68].

#### 3.1.5. Naringenin

It is known that naringenin possesses various pharmacologic properties including antioxidant, free radical scavenger, anticancer, anti-inflammatory, immunomodulator and memory enhancer.

It’s known that ICV-STZ administration at a sub-diabetogenic dose provided a relevant model for AD-type neurodegeneration with cognitive impairment (AD-TNDCI) [85,86]. In this field, Khan and collaborators (2012), investigated the effects of naringenin on cognitive dysfunction and, oxidative stress in a rat model of AD-TNDCI. The rats were orally pre-treated with naringin at 50 mg/kg for 2 weeks followed by ICV-STZ (3 mg/kg; 5 μL per site) injection bilaterally. The Authors observed that the imbalance of several markers of oxidative stress (enzymatic and non-enzymatic) with impairments in spatial learning and memory, loss of ChAT positive neuron and damage to hippocampal ones induced by ICV-STZ, were ameliorated by pre-treatment with naringenin [69]. The ability of naringenin to improve learning, memory and cognitive impairment was also confirmed by Baluchnejadmojarad and Roghani (2006) in an experimental model very similar to those described above [70].

Heo et al., (2004) examined the neuroprotective effect of naringenin found in *C. junos* against oxidative cell death induced by Aβ peptide in PC12 cells, and evaluated the anti-amnesic activity of naringenin using ICR mice with scopolamine-induced amnesia (1 mg/kg body weight). They showed that pretreatment with naringenin prevented the generation of Aβ-induced ROS and decreased Aβ toxicity in a concentration dependent manner. Furthermore, naringenin (4.5 mg/kg body weight), significantly ameliorated scopolamine-induced amnesia [71]. Vafeiadou and coworkers (2009) [73] demonstrated that naringenin (0.01–0.3 μmol/L) protected against LPS/IFN-gamma-induced neuronal death in a primary neuronal-glial co-culture system by inhibiting the p38 mitogen-activated protein kinase (MAPK) phosphorylation and downstream signal transducer and activator of transcription-1 (STAT-1).

More recently, Wu and coworkers (2016) demonstrated that naringenin inhibits the expression of cytokine signaling (SOCS)-3, iNOS and COX-2, as well as the release of NO and pro-inflammatory cytokines in microglial cells. These actions were modulated by adenosine monophosphate-activated protein kinase α (AMPKα) and protein kinase C δ (PKCδ) [72].

#### 3.1.6. Didymin

Only one paper suggested a neuroprotective effect of didymin that was found to increase cell viability of neuronal cells injured by H_2_O_2_ by decreasing mitochondrial dysfunctions and levels of intracellular ROS, stimulating SOD, CAT and GPx activity [74]. The mechanism underlying the protective effects of didymin in differentiated-SH-SY5Y exposed to H_2_O_2_ might be related to the activation of antioxidant defense enzymes as well as to the inhibition of apoptotic features such as p-JNK and caspase-3 [74].

### 3.2. Flavones

#### 3.2.1. Apigenin

Apigenin is a member of the flavone subclass of flavonoids present in fruits and vegetables. It has long been considered to have various biological activities such as antioxidant, antiinflammatory, anti-mutagenic and anti-tumorigenic properties. Apigenin has been shown to exert neuroprotective activity against endoplasmic reticulum stress-induced apoptosis in the HT22 murine hippocampal neuronal cells [87], in primary cultures of human neurons subjected to quinoloinic acid-induced excitotoxicity [88] and in glutamate-induced neurotoxicity in both murine cerebellar and cortical cell cultures [89].

Zhao et al. (2013) [90] reported the neuroprotective effects of the flavone in the amyloid precursor protein/presenilin 1 protein (APP/PS1) double transgenic AD mouse model. After feeding four month-old mice with apigenin (40 mg/kg) for 3 months, they observed both improvements in memory and learning deficits and reduction of fibrillar amyloid deposits. Additionally, the apigenin-treated mice showed restoration of the cortical extracellular signal-regulated protein kinase 1 (ERK)/ cAMP response element-binding protein (CREB)/BDNF pathway that is known to be involved in learning and memory typically affected AD patients. Finally, apigenin enhances both SOD and GPx activities [90]. Likewise, in Aβ_25–35_-induced amnesia mouse models, Liu et al. (2011) reported the capability of apigenin (20 mg/kg) to improve the spatial learning and memory and the neurovascular functionality [91]. Cognitive enhancing effects by apigenin (20 mg/kg, intraperitoneally) have been described also in an animal model in which the flavone delayed the long-term forgetting [92]. Another study performed by Taupin et al. (2009) highlighted that the administration of apigenin (25 mg/kg) for 10 days stimulated neurogenesis in the hippocampal region of the brain in 7-week old mice and resulted in improved performance in the Morris water maze [93]. Patil and its research group demonstrated that chronic intraperitoneally administration of apigenin (5–20 mg/kg) reversed cognitive deficits in aged and lipopolysaccharide (LPS)-intoxicated mice [94]. Additionally, apigenin improved motor skills and enhanced neurotrophic potential which has been reduced in 1-methyl-4-phenyl-1,2,3,6-tetrahydropyridine (MPTP)-induced Parkinsonism in mice [95]. Particularly, MPTP (25 mg/kg) was administrated for five consecutive days and then apigenin (10 and 20 mg/kg) was orally administrated for 26 days, including 5 days of pretreatment. After that, the Author performed behavioral study and biochemical estimation of oxidative stress biomarkers, observing (i) a reduced tyrosine hydroxylase (TH)-positive cells; (ii) a rise of BDNF amount and (iii) a decreased level of GFAP in the substantia nigra of MPTP-treated mice [95]. More recently, Liu et al. (2015) demonstrated that apigenin exerts a protective effect against MPP^+^-induced neurotoxicity in neuronal like catecholaminergic PC12 cells. This effect is mediated through the inhibition of oxidative stress, the stabilization of mitochondrial function and the reduction of neuronal apoptosis via the mitochondrial pathway [96]. The cytoprotective effect of apigenin was also evaluated by Wu and coworkers (2015), showing that apigenin restored cell viability and repressed both caspase-3 and PARP-1 activation in 4-HNE-treated PC12 cells. Moreover, apigenin activated MAPK and Nrf2 signaling, which in turn evoked adaptive cellular stress response pathways, restored ER homeostasis altered by 4-HNE and inhibited cytotoxicity [97].

Table 2 reported the studies in which was evaluated apigenin as potential neuroprotective agent.

#### 3.2.2. Luteolin

In the last decades, several studies reported the neuroprotective properties of luteolin [111]. They are summarized in Table 2. In vivo studies showed that luteolin protected against cognitive dysfunction induced by chronic cerebral hypoperfusion in rats [98,99]. Luteolin also protected against high fat diet-induced cognitive defects in obesity mice [100]. Recently, Wang et al., (2016) demonstrated that the flavone significantly ameliorated the spatial learning and memory impairment and increased the thickness of CA1 pyramidal layer in STZ-treated animals [101]. Moreover, luteolin (20 μM) can attenuate 6-OHDA-caused oxidative stress, cytotoxicity, and caspase-3 activation in PC12 cells [102]. Another study displayed as luteolin (10 and 20 mg/kg) could improve locomotor and muscular alterations in mice exposed to MPTP, also decreasing the TH-positive cells, the neurotrophic factors, the GFAP, and the BDNF [95]. In addition, Wruck et al., (2007) demonstrated that luteolin protected rat neural PC12 and glial C6 cells from MPP^+^-induced toxicity and activated Nrf2 [103]. The research group of Rezai-Zadeh (2008) demonstrated that treatment of both N9 and murine-derived primary microglia cell lines with luteolin significantly reduced both the IFN-γ-induced CD40 expression and the release of pro-inflammatory cytokines IL-6 and TNF-α through the inactivation of STAT1 [104]. One year later, the same authors showed that luteolin treatment of murine N2a cells transfected with SweAPP and primary neuronal cells derived from SweAPP overexpressing mice resulted in a significant reduction in Aβ generation. The mechanism seems to be involved in the selective inactivation of the GSK-3α isoform, which in turn increases the phosphorylation of PS1, the catalytic core of the γ secretase complex, thereby reducing PS1 APP interaction and Aβ generation [105].

Excessive production of NO and pro-inflammatory cytokines by activated microglia plays a pivotal role in the pathogenesis of ND. Kao and collaborators (2011) reported the inhibitory effect of luteolin on LPS/interferon γ (IFN-γ)-induced NO and cytokines production in rat primary microglia and BV-2 microglial cells. Particularly, luteolin concentration-dependently abolished LPS/IFN-γ-induced NO, TNF-α and IL-1β production as well as iNOS expression. Luteolin also exerted inhibitory effect on transcription factor activity including NF-κB, signal transducer and activator of transcription 1 (STAT1) and interferon regulatory factor 1 (IRF-1). These effects were accompanied by down-regulation of ERK, p38, JNK, protein kinase B (Akt) and Src [106].

Chen and coworkers (2008) demonstrated that luteolin may protect dopaminergic neurons from LPS-induced injury and its efficacy in inhibiting microglia activation [107]. Another research showed that luteolin inhibited the LPS-stimulated expression of inducible iNOS, COX-2, TNF-α and IL-1β as well as blocked the LPS-induced NF-κB activation [108]. A few years later, the same authors, demonstrated that luteolin inhibited the LPS-stimulated expression of TLR-4, blocked LPS-induced NF-κB, p38, JNK and Akt activation, but had no effect on ERK. In addition, pre-treatment with luteolin increased cell viability and reduced apoptosis of SH-SY5Y cells co-cultured with LPS-stimulated BV2 microglia [109]. To better understand the immuno-modulatory effects of luteolin, Dirscherl et al., (2010) carried out a genome-wide expression study in LPS-exposed BV-2 microglia cells treated with luteolin and performed a phenotypic and functional profile. They observed that luteolin suppressed pro-inflammatory marker expression in LPS-activated microglia and triggered global changes in the microglial transcriptome with more than 50 differentially expressed transcripts. Moreover, pro-inflammatory and pro-apoptotic gene expression was blocked by luteolin, while mRNA levels of genes involved into anti-oxidant metabolism, phagocytosis, ramification, and chemotaxis were significantly induced [110].

### 3.3. Flavonols

#### 3.3.1. Kaempferol

There are few evidences for the neuroprotective effect of kaempferol (Table 3). LPS-activated microglial cells have been suggested to be a useful in vitro model to test the potential of drugs for neuroinflammatory disorders [112,113]. Park and coworkers (2011) demonstrated the activity of kaempferol against neuroinflammatory toxicity caused by LPS-activated microglia. Particularly, they showed that kaempferol inhibit the LPS-induced expression of iNOS, COX-2, matrix metalloproteinases (MMPs) and the subsequent production of ROS, TNF-α, NO, PGE2 and IL-1β in BV2 microglial cells, through the inhibition of TLR4, NF-κB, p38 MAPK, JNK and AKT activation [114]. Yang and collaborators (2014) demonstrated the neuroprotective effects of kaempferol in glutamate-treated hippocampal neuronal cells, suggesting that kaempferol may be a useful candidate for neurodegenerative diseases [115].

#### 3.3.2. Rutin

Rutin is a multifunctional flavonoid glycoside acting on various cellular functions under pathological conditions such as ND, maybe due to its ability to cross the blood brain barrier (BBB) [126]. Table 3 summarizes the studies aimed to evaluate the effects of rutin in models of neurodegenerations. In an in vitro model of PD, it has been demonstrated that rutin reduced lipid peroxidation in 6-OHDA-treated PC-12 cells, activating antioxidant enzymes like SOD, CAT, GPx and GSH [116]. The neuroprotective effects of rutin against 6-OHDA were evaluated also in vivo. Rats was pre-treated with this flavonoids (25 mg/kg body weight, orally) for 3 weeks and then subjected to unilateral intrastriatal injection of 6-OHDA (10 μg in 0.1% ascorbic acid). Rutin prevented the deficits in locomotor activity and motor coordination, and protected neurons from deleterious effects of 6-OHDA in the substantia nigra, as suggested by histopathological and immunohistochemical assays [121].

Oxidative stress has been proposed to be a potential mechanism underlying ethanol-induced damage and may contribute to neuronal degeneration [127]. Song et al., (2014) investigated the antioxidant effect of rutin in hippocampal neuronal cells exposed to ethanol, and found that it prevented the ethanol-induced decrease in nerve growth factor expression, GDNF and BDNF in HT22 cells. Moreover, rutin significantly increased the level of the antioxidant glutathione, and the activities of SOD and CAT [117]. Oxidative stress may play a role also in hippocampal cell death associated with KA-induced neurotoxicity [128]. It has been reported that rutin (100 and 200 mg/kg, i.p. for 7 days) has potential anticonvulsant and antioxidative activities against oxidative stress in KA-induced (10 mg/kg, i.p.) seizures in mice [120].

Wang and coworkers (2012) showed that rutin can inhibit Aβ_42_ fibrillization in concentration dependent manner and can attenuate Aβ_42_-induced cytotoxicity in SH-SY5Y neuroblastoma cells [122]. More recently, the same authors demonstrated that orally administered rutin significantly attenuated memory deficits in APPswe/PS1dE9 transgenic mice, decreased oligomeric Aβ level, reduced oxidative stress, downregulated gliosis and diminished IL-1 and IL-6 levels in the brain [123]. Another study suggested a role for rutin in protecting against AD. Moghbelinejad and coworkers (2014) showed that rutin improved memory retrieval in rats injected with Aβ by increasing ERK1, CREB and BDNF [125].

Recently, Yu et al., (2015) showed that rutin inhibited amylin-induced neurocytotoxicity, decreasing the production of ROS, NO, glutathione disulfide (GSSG), MDA, TNF-α and IL-1β, thus attenuating mitochondrial damage and increasing the GSH/GSSG ratio. These protective effects could be derived by its ability to inhibit amylin aggregation, enhance the activity of SOD, CAT, and GPx, and reduce that of iNOS [118]. Machawal and Kumar (2014) suggested that the neuroprotective mechanism of rutin against immobilization stress-induced anxiety-like behavioral and oxidative damage in mice is mediated by a reduction of NO [124]. Finally, it has been proposed that rutin protects against the neurodegenerative effects of prion accumulation in vitro, by reducing levels of ROS and NO, increasing production of neurotropic factors and inhibiting mitochondrial apoptotic events leading to HT22 neuronal cell death [119].

#### 3.3.3. Quercetin

Quercetin exhibits numerous pharmacological activities including anti-cancer, anti-inflammatory, anti-atherosclerotic, anti-thrombotic and anti-hypertensive effects, as well as benefits for human endurance exercise capacity. Several in vitro and in vivo studies have provided supportive evidence also for its neuroprotective effects in various models of neuronal injury and chronic neurodegenerative diseases (Table 4). In vitro studies have shown that low micromolar concentrations of quercetin inhibited cell toxicity induced by neurotoxic molecules known to be inducer of oxidative stress. In particular, several research employed H_2_O_2_ as stressor. Suematsu et al., (2011) demonstrated that quercetin suppressed the H_2_O_2_-caused cytotoxicity and inhibited apoptosis in human neuronal SH-SY5Y cells [129]. Quercetin also decreased H_2_O_2_-induced oxidative stress in SK-N-MC cells by reducing HIF-1a, Foxo-3a, NICD and pro-apoptotic mediators including p53 and Bax [130]. The anti-oxidative and anti-apoptotic role of quercetin was further supported by the study of Jazvinšćak Jembrek and coworkers (2012) [131]. Sajad et al., (2013) showed that pre-treatment with quercetin prevented protein nitration and glycolytic block of proliferation in cultured neuronal precursor cells (NPCs) [132].

Protection of neuronal cells from the toxicity of Aβ peptide-induced toxicity has also been reported. Ansari et al., (2009) showed that low concentration of quercetin significantly attenuated protein oxidation, lipid peroxidation and apoptosis induced by Aβ_1–42_ in neuronal cultures [133], while Zhang et al., (2016) demonstrated that quercetin enhanced brain apoE levels and decreased Aβ levels in the cortex of amyloid model mice [134]. Moreover, the orally administration of quercetin (50, 100 and 200 mg/kg body weight) markedly improves MPTP-induced dopamine depletion in the brain tissue, the motor balance and the coordination which is significantly altered following MPTP injection in an animal model of PD [135].

In 2005, Chen and collaborators, demonstrated that quercetin suppressed LPS- and IFN-γ-induced NO production and iNOS gene transcription and enhanced heme oxygenase-1 (HO-1) expression [136]. More recently, Kang and coworkers (2013) found that the suppression of NO system in BV2 microglial cell line is mediated by the inhibition of NF-κB and the induction of Nrf2/HO-1 [137]. Furthermore, quercetin protected neuronal PC12 cells from high-glucose-induced oxidation, nitrosative stress, and apoptosis [138], and antagonized cognitive impairment induced by feeding mice with a high fat [139] or high-cholesterol diet [140].

There is a lot of evidence showing that memory deficit is associated with impaired cerebral circulation and a decrease in the cholinergic system. It has been shown that quercetin protected the retina from apoptotic damage due to ischemia reperfusion injury in vivo [142] and was also neuroprotective in models of intracerebral hemorrhage in rats [141]. Tota et al., (2010) demonstrated that orally daily pre-treatment with quercetin (2.5, 5 and 10 mg/kg) showed a dose-dependent restoration of cerebral blood flow (CBF) and ATP content, significantly reduced by the administration of STZ (0.5 mg/kg) in mice. Further, quercetin prevented STZ-induced memory impairment and decreased AChE activity as well as oxidative and nitrosative stress, as evidenced by a significant decrease in MDA and nitrite, and increase in GSH levels [143]. Richetti et al., (2011) demonstrated the protective role of quercetin (single injection of 50 mg/kg concentration) against scopolamine-induced inhibitory avoidance memory deficits in zebrafish [144]. However, Nassiri-Asl (2013) observed that quercetin (50 mg/kg) could not be effective against oxidative stress in the hippocampus and cerebral cortex in kindled rats besides its anticonvulsant effects and protection against memory impairment [154].

Quercetin provided protection also against the neurotoxicity of several metals. Hu et al., (2008) evaluated the effect of quercetin on chronic lead (Pb) exposure-induced impairment of synaptic plasticity in dentate gyrus (DG) area of rat hippocampus. The results showed that quercetin significantly increase the depressed input/output (I/O) functions, paired-pulse reactions (PPR) and long-term potentiation (LTP) of Pb-exposed group. In addition, concentration of Pb in hippocampus was partially reduced after quercetin treatment [145]. Quercetin (0.5–50 mg/kg/bw/day) protected also against DNA damage caused by exposure to methylmercury [146] and tungsten [147]. Several lines of evidences suggested that Al has severe toxic manifestations on the CNS. The research group of Sharma, found that pre-treatment with quercetin (10 mg/kg/bw/day) before intragastrically administration of Al (10 mg/kg/bw/day) decreased ROS levels, mitochondrial DNA oxidation and citrate synthase activity in both hippocampus and corpus striatum regions, while increased MnSOD activity of rat brain. In addition, quercetin prevented Al-induced translocation of cytochrome c (cyt-c), up-regulated Bcl-2, and down-regulated Bax, p53 and caspase-3 activation. It also reduced DNA fragmentation and increased the mitochondrial DNA copy number and mitochondrial content in the regions of rat brain [148,149,150].

Polychlorinated biphenyls (PCBs) are very toxic environmental contaminants known to trigger neurochemical damages and behavioral disorders. Bavithra et al. (2012) showed that quercetin acted as scavenger of the PCBs-induced free radicals and protected dopaminergic receptors in the cerebellum of rat [151]. Another study carried out by Selvakumar and coworkers (2013), evidenced that quercetin (50 mg/kg) suppresses ROS, enhances both enzymatic antioxidants and neurotransmitter levels and improves the cognitive functions damned by PCBs (2 mg/kg) [152]. Moreover, quercetin prevented alterations on cholinergic neurotransmission and in the behavioral tests also in rats experimentally demyelinated by ethidium bromide [153].

### 3.4. Polymethoxiflavones

In Table 5 are summarized the studies reporting the neuroprotective activity of *Citrus* polymethoxiflavones.

#### 3.4.1. Tangeretin

Tangeretin is a polymethoxyflavone present exclusively in *Citrus* fruit peels. Neuroprotective effects of tangeretin were elucidated in an animal model of PD. Sub-chronic treatment of rats with tangeretin (20 mg/kg/day) for 4 days before 6-OHDA injection markedly reduced the loss of both TH^+^ cells and striatal dopamine content evoked by unilateral infusion of 6-OHDA (8 μg) onto medial forebrain bundle [155]. More recently, Shu and coworkers (2014) demonstrated that tangeretin suppressed microglial activation in the LPS-stimulated primary rat microglia and BV-2 cell culture [156] (Table 5).

#### 3.4.2. Nobiletin

Nobiletin is a polymethoxylated flavone that is commonly presents in *Citrus* peels. Several papers reported the protective effect of nobiletin in different model of AD. Matsuzaki and coworkers (2006) observed that nobiletin (10 and 30 μM) reversed a reduction in the activity of CREB-phosphorylation by the sublethal concentration of Aβ_1–42_ or Aβ_1–40_ in cultured rat hippocampus neurons. Moreover, it ameliorated Aβ_1–40_-induced impairment of memory in AD model rats [157].

Onozuka and collaborators (2008) demonstrated that daily administrated nobiletin (10 mg/kg, i.p.) for 4 months reduced Aβ plaque pathology and improved cognitive deficits in APP-SL 7–5 mice, a transgenic mouse model of AD [158]. More recently, the same authors showed that nobiletin (10–50 mg/kg/day, i.p.) improved age-related cognitive impairment of senescence-accelerated mouse prone 8 (SAMP8) mice, and reduced both the oxidative stress and tau phosphorylation in their brain [159]. Moreover, they showed that nobiletin (30 mg/kg) administered for 3 months counteracted the impairment of short-term memory and recognition memory in a triple transgenic mouse model of AD (3XTg-AD mice). In addition, nobiletin reduced the levels of both soluble Aβ_1–40_ and ROS in the brain and in the hippocampus of 3XTg-AD mice, respectively [160]. Furthermore, daily administration of nobiletin for four months rescued the memory impairment in fear conditioning, and decreased hippocampal Aβ deposit in the transgenic mice [161].

The effect of nobiletin on neurological disorders was also evaluated in a neurotoxic model of PD. MPP^+^ was unilaterally injected into the median forebrain bundle of rat brains with or without daily intraperitoneal injection of nobiletin (1, 10 and 20 mg/kg). The latter, at a dose of 10 mg/kg, but not at 1 or 20 mg/kg, significantly protected DA neurons in the substantia nigra of MPP^+^-treated rats, also reducing microglial activation [162]. The capability of nobiletin to suppress microgliosis was demonstrated also by the results of the study performed by Cui et al. (2010) [163]. They found that the polymethoxiflavone suppressed the activation of NF-κB signaling pathway and the release of NO, TNF-α and IL-1β, as well as the phosphorylations of ERK, JNK and p-38 evoked by LPS in BV-2 microglia cells.

It is known that decreased cerebral blood flow causes cognitive impairments and neuronal injury in the progressive age-related neurodegenerative disorders such as AD and vascular dementia. Yamamoto et al., (2009) showed that treatment with 50 mg/kg of nobiletin (i.p.) for the consecutive 7 days before and after the induction of brain ischemia significantly inhibited delayed neuronal death in the hippocampal CA1 neurons and improved the contextual cerebral ischemia-induced memory deficits [164]. Very recently, Zhang et al. (2016) demonstrated that nobiletin (10 and 25 mg/kg/day; i.p.) administrated for 3 days prior to the experimental ischemia and immediately after surgery reduced brain edema and neurological deficit, as well as increased the expression of Nrf2, HO-1, SOD1 and GSH, while decreased the levels of NF-κB, MMP-9 and MDA [165].

## 4. Conclusion Remarks and Future Perspectives

During the last decades, several studies were performed to evaluate the neuroprotective effects of *Citrus* flavonoids and to identify their molecular targets. Literature data collected in our review support their protective activity in neuropathological conditions, especially in the presence of prooxidants or neurotoxins. These findings highlight the antioxidant nature of flavonoids, able to arrest free radical-induced oxidative damage, which is known to play a pivotal role in many degenerative diseases. Moreover, their neuroprotective action is mediated by the interaction with specific intracellular targets that are implicated in several signaling pathways important for maintaining the physiological status. In this way, *Citrus* flavonoids prevent the neuronal dysfunction due to both acute and chronic injuries in several in vivo experimental models. Nevertheless, the majority of studies on the beneficial effects of *Citrus* flavonoids have been performed in vitro and in vivo, without evidence from equivalent clinical trials. This means that in some cases, the experimental research on the neuroprotective effects of *Citrus* flavonoids does not take into account some aspects which are of great importance in clinical practice. For example, in some study, the pharmacological effect was observed at concentrations or dosages (in vitro and in vivo studies, respectively) of active substance which are unlikely to be reached in the clinical setting. Moreover, in some study it was used routes of administration that highly unlikely to be used by humans. Generally, a natural drug is orally administered (often as nutraceutical or dietary supplement), but bioavailability and presystemic elimination issues are often ignored by researchers. Consequently, the beneficial effects of orally administered flavonoids to improve or prevent CNS pathologies still remain an uncompleted debated topic. This because their poor absorption, rapid metabolism and selective permeability across the BBB limits their access to the CNS and, consequently, their neuroprotective efficacy. In other words, we do not know if the exciting preclinical results correspond to a real therapeutic success. This is the reason for their limited clinical application both alone and as an add-on therapy. Nevertheless, the development of novel natural compound-loaded delivery systems has improved the bioavailability of flavonoids together with their delivery to the brain, enhancing the potential of flavonoids as neuroprotective agents. Further studies are necessary to unravel more pharmacokinetics aspects of flavonoids, which in turn would be very important to stimulate well designed and well-controlled clinical trials confirming the excellent preclinical results.

Anyway, as suggested by this literature revision, we can consider *Citrus* flavonoids as key compounds for the development of a new generation of pharmacological agents effective in preventing and treating neurodegenerative diseases.

This review may be used as a starting point for novel nutraceuticals, food supplements or complementary and alternative drugs to maintain or improve the neurophysiological status.

## Figures and Tables

**Table 1:** *Citrus* flavanones and experimental models of neurodegenerative diseases.

Flavanones	Model	Disease/Condition	References
**naringin**	In vivo	MPP^+^-treated rats	Leem et al., 2014 [51]
	In vivo	6-OHDA-injected mice	Kim et al., 2016 [52]
	In vivo	3-NP-injected rats	Gopinath and Sudhandiran, 2012 [53]
	In vivo	aluminum-treated rats	Prakash et al., 2013 [54]
	In vivo	colchicine-treated rats	Kumar et al., 2010 [55]
**hesperidin**	In vivo	6-OHDA-injected mice	Antunes et al., 2014 [56]
	In vivo	3-NP-treated mice	Menze et al., 2012 [57]
	In vivo	APP/PS1–21 transgenic mice	Li et al., 2015 [58]
	In vivo	APPswe/PS1dE9 transgenic mice	Wang et al., 2014 [59]
	In vivo	ICV-STZ-injected mice	Javed et al., 2015 [60]
	In vivo	AlCl_3_-injected rats	Thenmozhi et al., 2015 [61]
	In vivo	AlCl_3_-injected rats	Thenmozhi et al., 2016 [62]
	In vivo	4-AP-treated rats KA-injected rat	Chang et al., 2015 [63]
**hesperetin**	In vivo	Mice	Choi and Ahn, 2008 [64]
	In vivo	6-OHDA-injected rats	Kiasalari et al., 2016 [65]
	In vitro	staurosporine-treated cortical neurons cultures	Rainey-Smith et al., 2008 [66]
	In vitro	H_2_O_2-_treated cortical neurons	Vauzour et al., 2007 [67]
**neohesperidin**	In vitro	H_2_O_2_-treated PC12 cells	Hwang et al., 2008 [68]
**naringenin**	In vivo	ICV-STZ-injected rats	Khan et al., 2012 [69]
	In vivo	ICV-STZ-injected rats	Baluchnejadmojarad and Roghani, 2006 [70]
	In vitro	Aβ-treated PC12 cells	Heo et al., 2004 [71]
	In vitro	microglial cells	Wu et al., 2016 [72]
	In vitro	LPS/IFN-γ exposed primary neuronal-glial cells	Vafeiadou et al., 2009 [73]
**didymin**	In vitro	H_2_O_2-_treated neuronal cells	Morelli et al, 2014 [74]

**Table 2:** Studies employed the *Citrus* flavones and their experimental models of neurodegeneration.

Flavones	Model	Disease/Condition	Reference
**apigenin**	In vitro	HT22 cells	Choi et al., 2010 [87]
	In vitro	QUIN-treated primary human neuron	Braidy et al., 2010 [88]
	In vitro	glutamate-treated cerebellar and cortical neurons	Losi et al., 2004 [89]
	In vivo	APP/PS1 double transgenic mouse	Zhao et al., 2013 [90]
	In vivo	Aβ_25–35_-treated mouse	Liu et al., 2011 [91]
	In vivo	young male Wistar rats	Popovic et al., 2014 [92]
	In vivo	7-week old mice	Taupin et al., 2009 [93]
	In vivo	MPTP-treated mice	Patil et al., 2014 [95]
	In vitro	MPP^+^-incubated PC12 cells	Liu et al., 2015 [96]
	In vitro	4-HNE-exposed PC12 cells	Wu et al., 2015 [97]
	In vivo	aged and LPS-intoxicated mice	Patil et al., 2003 [94]
**luteolin**	In vivo	chronic cerebral hypoperfusion in rats	Fu et al., 2014 [98]
	In vivo	chronic cerebral hypoperfusion in rats	Xu et al., 2010 [99]
	In vivo	obesity mice	Liu et al., 2014 [100]
	In vivo	ICV-STZ injected rat	Wang et al., 2016 [101]
	In vitro	6-OHDA-exposed PC12 cells	Hu et al., 2014 [102]
	In vivo	MPTP-treated mice	Patil et al., 2014 [95]
	In vitro	MPP^+^-exposed PC12 and glial C6 cells	Wruck et al., 2007 [103]
	In vitro	IFN-γ-incubated N9 and microglia cells	Rezai-Zadeh et al., 2008 [104]
	In vitro	N2a cells transfected with SweAPP	Rezai-Zadeh et al., 2009 [105]
	In vitro	LPS/IFN-γ-treated rat primary microglia and BV-2 microglial cells	Kao et al., 2011 [106]
	In vitro	LPS-incubated microglia cells	Chen et al., 2008 [107]
	In vitro	LPS-exposed BV2 microglia	Zhu et al., 2011 [108]
	In vitro	SH-SY5Y cells co-cultured with LPS-stimulated BV2 microglia	Zhu et al., 2014 [109]
	In vitro	LPS-treated microglia cells	Dirscherl et al., 2010 [110]

**Table 3:** *Citrus* flavonols kaempferol and rutin in models of neurodegenerations.

Flavonols	Model	Disease/Condition	Reference
**kaempferol**	In vitro	LPS-activated microglia	Park et al., 2011 [114]
	In vitro	glutamate-treated hippocampal neuronal	Yang et al., 2014 [115]
**rutin**	In vitro	6-OHDA-incubated PC-12 cells	Magalingam et al., 2013 [116]
	In vitro	ethanol-exposed HT22 cells	Song et al., 2014 [117]
	In vitro	amylin-treated SH-SY5Y cells	Yu et al., 2015 [118]
	In vitro	neuronal cells	Na et al., 2014 [119]
	In vivo	KA-injected BALB/c mice	Nasiri-Asl et al., 2013 [120]
	In vivo	6-OHDA-treated rats	Kham et al., 2012 [121]
	In vitro	Ab_42_-incubated SH-SY5Y cells	Wang et al., 2012 [122]
	In vivo	APPswe/PS1dE9 transgenic mice	Xu et al., 2014 [123]
	In vivo	mice	Machawal and Kumar, 2014 [124]
	In vivo	Aβ-injected rats	Moghbelinejad et al., 2014 [125]

**Table 4:** Studies employed quercetin in experimental models of neurodegeneration.

Flavonol	Model	Disease/Condition	Reference
**quercetin**	In vitro	H_2_O_2_-incubated SH-SY5Y cells	Suematsu et al., 2011 [129]
	In vitro	H_2_O_2_-exposed SK-N-MC cells	Roshanzamir and Yazdanparast, 2014 [130]
	In vitro	H_2_O_2_-treated NPCs cells	Sajad et al., 2013 [132]
	In vitro	H_2_O_2_-treated -P19 neurons	Jazvinšćak J et al., 2012 [131]
	In vitro	neuronal cells	Ansari et al., 2009 [133]
	In vivo	Aβ-injected mice	Zhang et al., 2016 [134]
	In vivo	MPTP-treated mice	Lv et al., 2012 [135]
	In vitro	LPS- and IFN-γ-treated BV-2 microglia	Chen et al., 2005 [136]
	In vitro	LPS-treated BV-2 microglia	Kang et al., 2013 [137]
	In vitro	high-glucose exposed PC12 cells	Bournival et al., 2012 [138]
	In vivo	high-fat diet in mice	Xia et al., 2015 [139]
	In vivo	high-cholesterol diet in mice	Lu et al., 2010 [140]
	In vivo	intracerebral hemorrhage in rats	Zhang et al., 2015 [141]
	In vivo	ischemia reperfusion injury in rats	Arikan et al., 2015 [142]
	In vivo	STZ-treated mice	Tota et al., 2010 [143]
	In vivo	scopolamine-treated zebrafish	Richetti et al., 2011 [144]
	In vivo	Pb-exposed rats	Hu et al., 2008 [145]
	In vivo	ethylmercury-exposed rats	Barcelos et al., 2011 [146]
	In vivo	tungsten-exposed rats	Sachdeva et al., 2015 [147]
	In vivo	Al-treated rats	Sharma et al., 2013 [148]
	In vivo	Al -treated rats	Sharma et al., 2015 [149]
	In vivo	Al -treated rats	Sharma et al., 2016 [150]
	In vivo	PCBs-treated rats	Bavithra et al., 2012 [151]
	In vivo	PCBs-treated rats	Selvakumar et al., 2013 [152]
	In vivo	ethidium bromide-treated rats	Beckmann et al., 2014 [153]

**Table 5:** Studies and experimental models of neurodegenerative diseases in which tangeretin or nobiletin were used.

Polymethoxyflavones	Model	Disease/Condition	Reference
**tangeretin**	In vivo	6-OHDA-injected rat	Datla et al., 2001 [155]
	In vitro	LPS-stimulated microglia and BV-2 cells	Shu et al., 2014 [156]
**nobiletin**	In vitro	Aβ_1–42_ or Aβ_1–40_-exposed hippocampus neurons of rats	Matzukazi et al., 2006 [157]
	In vivo	Aβ_1–40_-treated rats	
	In vivo	APP-SL 7-5 mice	Onozuka et al., 2008 [158]
	In vivo	SAMP8 mice	Nakajima et al., 2013 [159]
	In vivo	3XTg-AD mice	Nakajima et al., 2015 [160]
	In vivo	transgenic mice	Yamakuni et al., 2010 [161]
	In vivo	MPP^+^- injected mice	Jeong et al., 2014 [162]
	In vitro	LPS-treated BV-2 microglia cell	Cui et al., 2010 [163]
	In vivo	cerebral ischemia inducted mice	Yamamoto et al., 2009 [164]
	In vivo	cerebral ischemia inducted rats	Zhang et al., 2016 [165]
